# Arabidopsis ASYMMETRIC LEAVES2 protein required for leaf morphogenesis consistently forms speckles during mitosis of tobacco BY-2 cells via signals in its specific sequence

**DOI:** 10.1007/s10265-012-0479-5

**Published:** 2012-02-17

**Authors:** Lilan Luo, Sayuri Ando, Michiko Sasabe, Chiyoko Machida, Daisuke Kurihara, Tetsuya Higashiyama, Yasunori Machida

**Affiliations:** 1 Division of Biological Science, Graduate School of Science, Nagoya University, Chikusa-ku, Nagoya, 464-8602 Japan; 2 Graduate school of Bioscience and Biotechnology, Chubu University, 1200 Matsumoto-cho, Kasugai, Aichi 487-8501 Japan; 3 JST ERATO Higashiyama Live-Holonics Project, Nagoya University, Furo-cho, Chikusa-ku, Nagoya, Aichi 464-8602 Japan

**Keywords:** Arabidopsis, AS2 protein, Cell division, Leaf development, Nuclear speckles, Subnuclear localization

## Abstract

Leaf primordia with high division and developmental competencies are generated around the periphery of stem cells at the shoot apex. Arabidopsis ASYMMETRIC-LEAVES2 (AS2) protein plays a key role in the regulation of many genes responsible for flat symmetric leaf formation. The *AS2* gene, expressed in leaf primordia, encodes a plant-specific nuclear protein containing an AS2/LOB domain with cysteine repeats (C-motif). AS2 proteins are present in speckles in and around the nucleoli, and in the nucleoplasm of some leaf epidermal cells*.* We used the tobacco cultured cell line BY-2 expressing the AS2-fused yellow fluorescent protein to examine subnuclear localization of AS2 in dividing cells. AS2 mainly localized to speckles (designated AS2 bodies) in cells undergoing mitosis and distributed in a pairwise manner during the separation of sets of daughter chromosomes. Few interphase cells contained AS2 bodies. Deletion analyses showed that a short stretch of the AS2 amino-terminal sequence and the C-motif play negative and positive roles, respectively, in localizing AS2 to the bodies. These results suggest that AS2 bodies function to properly distribute AS2 to daughter cells during cell division in leaf primordia; and this process is controlled at least partially by signals encoded by the AS2 sequence itself.

**Electronic supplementary material:**

The online version of this article (doi:10.1007/s10265-012-0479-5) contains supplementary material, which is available to authorized users.

## Introduction

By means of the coordinated control of cell differentiation and proliferation, leaves develop from the peripheral zone of the shoot apical meristem (SAM) with indeterminate stem cells. Initially, a group of cells, which might be in a determinate state, is generated along the proximal–distal axis. Subsequently, the adaxial-abaxial axis in early-stage leaf primordia is established for further leaf development, as cells proliferate along the medial-lateral axis, and results in flat symmetrical leaves (Steeves and Sussex 1989; Hudson 2000; Tsukaya 2006; Szakonyi et al. 2010).

In *Arabidopsis thaliana,* several members of the class III homeodomain-leucine zipper (HD-ZIPIII) gene family determine adaxial cell fate (McConnell and Barton 1998; McConnell et al. 2001; Emery et al. 2003) and are negatively regulated by microRNAs (Bao et al. 2004; Mallory et al. 2004). Members of the *YABBY* (*YAB*) and the *KANADI* (*KAN*) gene families are involved in the specification of abaxial cell fate in the leaf lamina (Sawa et al. 1999; Siegfried et al. 1999; Bowman and Smyth 1999; Kerstetter et al. 2001; Eshed et al. 2001, 2004; Kumaran et al. 2002; Wu et al. 2008; Sarojam et al. 2010). In addition, *ETTIN*/*AUXIN RESPONSE FACTOR3* (*ETT*/*ARF3*) and *ARF4* specify both abaxial cell fate and lateral growth of leaves (Pekker et al. 2005)*.* Transcripts of these genes are down-regulated by a *trans*-acting small interfering RNA in the adaxial domain of leaf primordia (Montgomery et al. 2008; Chitwood et al. 2009; Schwab et al. 2009). Although these effector genes required for the establishment of polarity have been identified, the system controlling the expression of such effector genes remains to be identified.

The *ASYMMETRIC LEAVES1* (*AS1*) and *AS2* genes of *A. thaliana* are involved in the formation of appropriately expanded and flat symmetrical leaves (Rédei and Hirono 1964; Tsukaya and Uchimiya 1997; Byrne et al. 2000; Ori et al. 2000; Semiarti et al. 2001; Iwakawa et al. 2002). Mutations in these genes are associated with pleiotropic abnormalities in leaves observed along the three developmental axes described above. AS1 and AS2 proteins form a complex (Xu et al. 2003; Yang et al. 2008), hereinafter referred to as AS2/AS1. In leaf primordia, AS2/AS1 represses both the expression of genes for such abaxial determinants as *ETT/ARF3* (Iwakawa et al. 2007; Takahashi et al. 2008) and the expression of class 1 *KNOTTED*-like homeobox (*KNOX*) genes, such as *BREVIPEDICELLUS* (*BP*), which are normally expressed in the SAM and its periphery and apparently function in maintaining the indeterminate cell state (Long et al. 1996; Ori et al. 2000; Byrne et al. 2000; 2002; Semiarti et al. 2001; Lin et al. 2003). In addition, AS2/AS1 directly represses the transcription of *BP* and *KNAT2* by binding to their 5′-upstream regions (Guo et al. 2008). Some of the pleiotropic abnormalities of *as2* and *as1* plants, such as short leaves and decreases in the efficiency of root regeneration, have been attributed to the ectopic expression of class 1 *KNOX* genes (Ikezaki et al. 2010). Recently, Ishibashi et al. (2012) showed that enhanced expression of the *ETT/ARF3* gene in the *as2* mutant is responsible for less efficient adaxialization and asymmetric leaf lamina in *as2* (and also *as1*). Thus, AS1/AS2 participates in repressing the expression of class 1 *KNOX* and *ARF* genes to form expanded and flat symmetric leaves; however, the means by which *KNOX* and *ARF* gene expression is controlled by AS1/AS2 remains to be elucidated.

Both *AS1* and *AS2* genes encode nuclear proteins and are expressed in cells having high cell-division competence. *AS1*, which is expressed mainly around vascular tissues of cotyledonary nodes and leaf primordia (Iwakawa et al. 2007), encodes a myb-domain protein (Byrne et al. 2000). *AS2* is expressed mainly in the adaxial domain of embryonic cotyledons and leaf primordia and encodes a plant-specific protein having an AS2/LOB domain near the amino terminus (N-terminus) that consists of cysteine repeats (the C-motif) (Iwakawa et al. 2002; Shuai et al. 2002; Matsumura et al. 2009). In addition, AS2 protein is present in subnuclear bodies in and around the nucleoli as well as the nucleoplasm in some epidermal cells of *A. thaliana* leaves (Ueno et al. 2007). AS1 proteins are also present in subnuclear bodies, some of which co-localize to the bodies formed by AS2 (Ueno et al. 2007; Zhu et al. 2008). Investigation of the molecular and cellular bases behind the characteristic localization of AS2 protein should be one of the tactically available approaches for understanding the molecular mechanism of gene expression that is regulated by AS2 (also AS1).

In the present study, we investigated sub-nuclear localization of the AS2-fused yellow fluorescent protein (YFP) (AS2-YFP) in the tobacco cultured cell line BY-2, which is considered to be a typical and highly proliferative cell line. We observed that subnuclear speckles showing the YFP signal were present in only a limited portion of BY-2 interphase cells, whereas such speckles were seen in almost all cells undergoing mitosis, with distribution patterns that do not seem to be stochastic. We then performed deletion analysis of the AS2 sequence to seek for signal sequences required for the localization to the speckles. Here, we report our results showing that two short stretches of the AS2 sequence including the C-motif play critical roles in the localization of AS2 to the speckles.

## Materials and methods

### Construction of plasmids carrying the *AS2* sequence and its derivatives

To express YFP fusions in cells, full-length *AS2* cDNA and its truncated cDNA fragments, which are shown in Fig. 2a, were PCR-amplified with specific primer pairs (Table S1 and Fig. S1) and cloned into YFP fusion vector pEYFP (CLONTECH, Mountain view, CA, USA). Structures of all constructs were verified by sequencing. The resulting *AS2*-*EYFP* and truncated *AS2*-*EYFP* cDNA fragments were subcloned into the binary vector pER8 (Zuo et al. 2000).

### Cell culture and transformation

The tobacco cell line BY-2 was maintained in suspension culture at 26°C in the dark with weekly subculturing in modified Linsmaier and Skoog medium (Banno et al. 1993). Transformed BY-2 cells were generated by *Agrobacterium*-mediated transformation (An 1985).

### Chemicals and induction of transgene expression

17-β-estradiol was purchased from Sigma (St. Louis, MO, USA), prepared as a 20 mM stock solution in dimethyl sulfoxide (DMSO), and stored at −20°C in small aliquots. DMSO alone had no effect on transgene expression (data not shown). Independent transformants were separately subcultured in liquid LS medium containing 0.05 μM 17-β-estradiol for 16 h at 26°C in the dark. Several lines expressing YFP fusions were selected and analyzed.

### Fluorescence microscopy

For DAPI staining, cells were fixed with 3.7% paraformaldehyde in sodium phosphate buffer (pH 7.2) for 15 min and stained with 0.2 μg/ml of 4′,6-diamidino-2-phenylindole (DAPI). Images were recorded by confocal microscopy with a 40 × 1.3 NA plan apochromat oil immersion objective (FV1000 laser scanning microscope, OLYMPUS, Japan).

## Results

### AS2 bodies are detected in BY-2 cells undergoing progression of M phase and a limited proportion of cells at interphase of the cell cycle

**Fig. 1:**
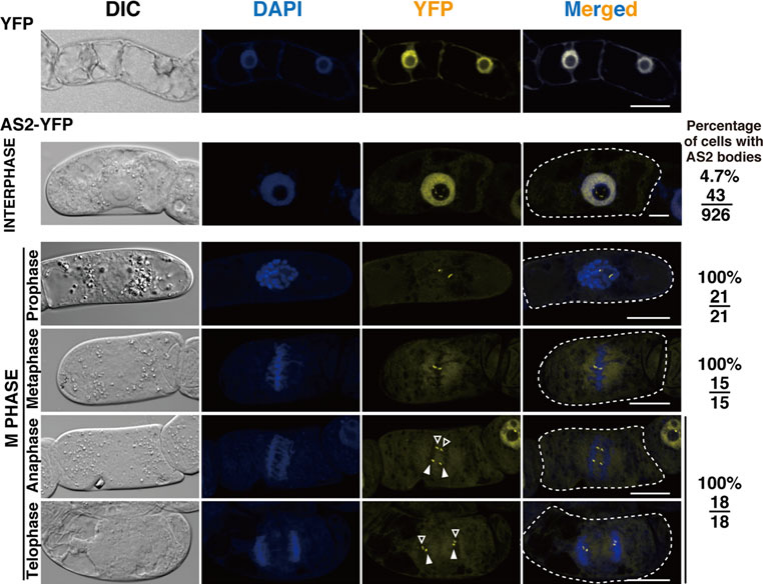
Subcellular localization of AS2-YFP in BY-2 cells at interphase and during mitosis. Expression of *AS2*-*YFP* and *YFP* is controlled under the estrogen-inducible promoter. Transformed BY-2 cell lines harboring *AS2*-*YFP* and *YFP* constructs were incubated for 16 h in the presence of 0.05 μM 17-β-estradiol. Cells were fixed and stained with 4′6-diamidino-2-phenylindole (DAPI), which is specific for nuclei. Fluorescence of DAPI (*blue*) and fluorescence of YFP (*yellow*) in BY-2 cells at the different cell cycle stages are visualized by using confocal fluorescence microscopy. Merged images (*blue* DAPI, *yellow* YFP) are shown on the *right* (Merged). Nomarski images are also shown (DIC). *Numbers on the right* represent ratios of cells showing AS2 bodies to total cells examined at specific phases of the cell cycle. In anaphase and telophase cells, AS2 bodies that seem to belong to the same pair are marked with a common shape of *arrowheads* or *arrows*. *Bars* 20 μm

We made the *XVE:AS2*-*YFP* DNA construct, in which the coding sequence of *AS2* cDNA was fused in frame to the coding sequence for yellow fluorescent protein (YFP). This fusion gene was driven by the estrogen-inducible promoter (Zuo et al. 2000) and introduced into cells of the tobacco cultured-cell line BY-2. As described in “Materials and methods”, we cultured the transformed BY-2 cells in liquid medium containing 0.05 μM 17-β-estradiol for 16 h to induce transcription of the *AS2*-*YFP* gene. We then examined those cells for subcellular and subnuclear localization of AS2-YFP proteins by observing randomly dividing cells for the fluorescent signals of AS2-YFP. As shown in Fig. 1, 4.7% of interphase cells exhibited YFP signals in subnuclear speckles in and around the nucleolus in addition to the nucleoplasm (Fig. 1, 2nd row). When only YFP was expressed in BY-2 cells (Fig. 1, 1st row), the signal was detected mainly in the nucleoplasm, as well as cytoplasm, similarly as was seen in the remaining cells at interphase with AS2-YFP (data not shown).

A small proportion (5.5%) of the culture of randomly dividing transformed BY-2 cells underwent mitosis (Fig. 1). Signals from AS2-YFP were detected in several concentrated speckles in all mitotic cells (prophase to telophase in Fig. 1). Interestingly, these speckles were observed to localize in pairwise patterns adjacent to both separating sets of daughter chromosomes after metaphase (Fig. 1, 5th and 6th rows: each pair is marked by the same symbol). There were no sets of daughter chromosomes that were not accompanied by the speckles, even when only a small number of the speckles were present in the parental nuclei (see also Fig. 3). The speckles appeared to be localized to regions among condensed chromosomes in the cells at prophase. From metaphase to telophase, however, the speckles seem to locate in peripheral regions of the chromosomes. Since the signals of AS2-YFP (seen in yellow in Fig. 1) in these phases were distinct from those of DAPI (seen in blue), speckles were not always associated with segregating daughter chromosomes. The numbers and sizes of speckles were variable from cell to cell, although most of the cells at interphase and in mitosis contained 2–6 speckles (data not shown). The number of speckles in mitotic cells was also variable, but interestingly the average numbers at metaphase and anaphase-telophase were 2.0 (81 speckles in 41 cells) and 3.8 (68 speckles in 18 cells), respectively.

In summary, patterns of subnuclear localization of the AS2 protein were dynamically altered during progression of the cell division cycle of BY-2 cells. AS2 was consistently incorporated into several speckles in cells undergoing mitosis and also in a small portion of cells at interphase; conversely, AS2 was also dispersed in the nucleoplasm of almost all cells at interphase. We hereinafter refer to these speckles in BY-2 cells as AS2 bodies.

### The short amino-terminal region of AS2 protein plays a negative role in the formation of AS2 bodies in interphase cells

**Fig. 2:**
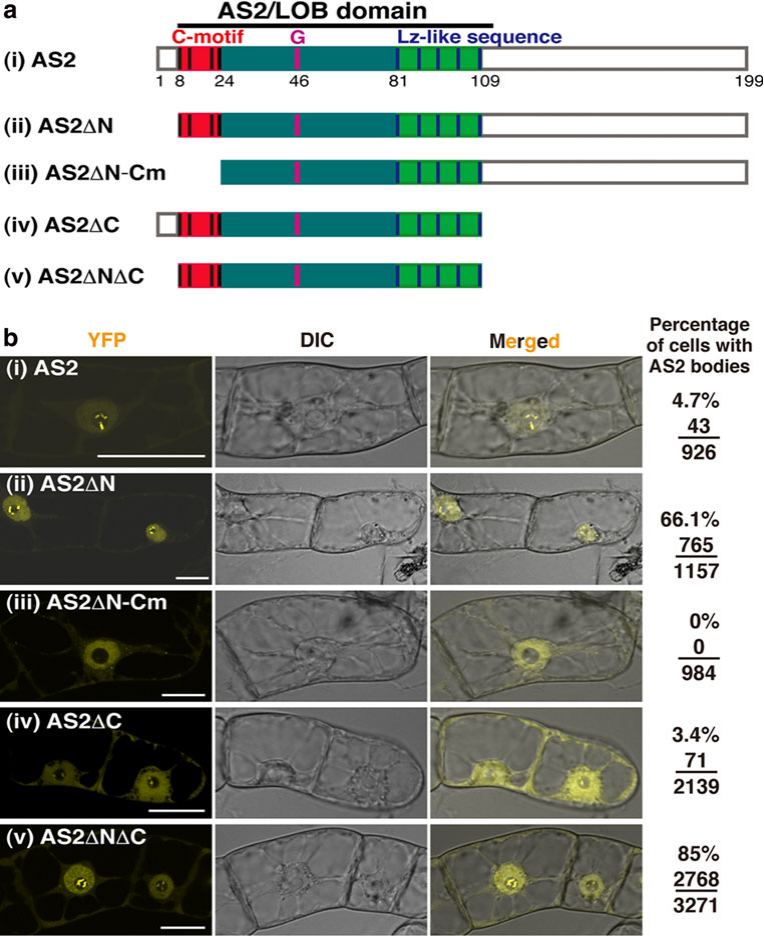
Subcellular localization of wild-type AS2 and its deletion mutants that were fused to YFP. **a** Schematic representation of wild-type and deletion mutants. Predicted domain organization and relevant amino-acid positions for AS2 are indicated *above* and *below*, respectively, in the wild-type schematics. Coding sequences for all AS2 proteins (i–v) were fused to the sequence corresponding to the N-terminus of the YFP sequence. These fusion constructs were linked to the estrogen-inducible promoter. **b** Subcellular localization of deletion mutants of AS2 in interphase cells of transformed BY-2 lines. The transformed cells harboring these fusion constructs were incubated for 16 h in the presence of 0.05 μM 17-β-estradiol. Living cells were observed by confocal fluorescence microscopy to detect fluorescence of YFP (*yellow* YFP). Nomarski (DIC) and merged images (Merged) are also shown. *Numbers on the right* represent ratios of cells showing AS2 bodies to interphase cells examined. *Bars* 20 μm

To seek for internal regions of the AS2 protein that might be responsible for its sub-nuclear localization patterns, we introduced a series of deletions into the AS2 moiety in AS2-YFP (Fig. 2a) and investigated the localization patterns of signals from the YFP fusions (Fig. 2b). As shown in Fig. 2b(ii), 66.1% of cells that expressed AS2ΔN-YFP (Fig. 2a(ii)), which had a deletion of the amino-terminal (N-terminal) short sequence (from residues 1 to 7), exhibited YFP signals in AS2 bodies during interphase. Thus, the proportion of cells that generate AS2 bodies increased markedly, suggestive of a negative role for the N-terminal sequence of AS2 protein in the formation of the AS2 bodies during interphase.

When the deleted region was extended into the C-motif, which is perfectly conserved in all members of the AS2/LOB family (Fig. 2a(iii)), signals from AS2ΔN-Cm-YFP were observed in the nucleoplasm in all randomly dividing BY-2 cells at interphase (Fig. 2b(iii)), which suggests that the C-motif is required for the localization of AS2 protein to AS2 bodies.

We deleted the carboxy-terminal (C-terminal) domain of AS2 in the AS2-YFP fusion (Fig. 2a(iv): designated AS2ΔC). Figure 2b(iv) shows that YFP signals in only a small portion of the interphase cells (3.4%) were visible in AS2 bodies. These results demonstrate that the C-terminal domain is not involved in the formation of AS2 bodies in interphase. Signals from AS2ΔNΔC-YFP that contained only the AS2/LOB domain sequence (Fig. 2a(v), b(v)), which is highly conserved in all members of the AS2/LOB family, were localized to AS2 bodies in 85% of cells at interphase, showing that the AS2/LOB domain is sufficient for the AS2 body formation.

### The C-motif in the AS2/LOB domain is essential for the formation of AS2 bodies during mitosis in BY-2 cells

**Fig. 3:**
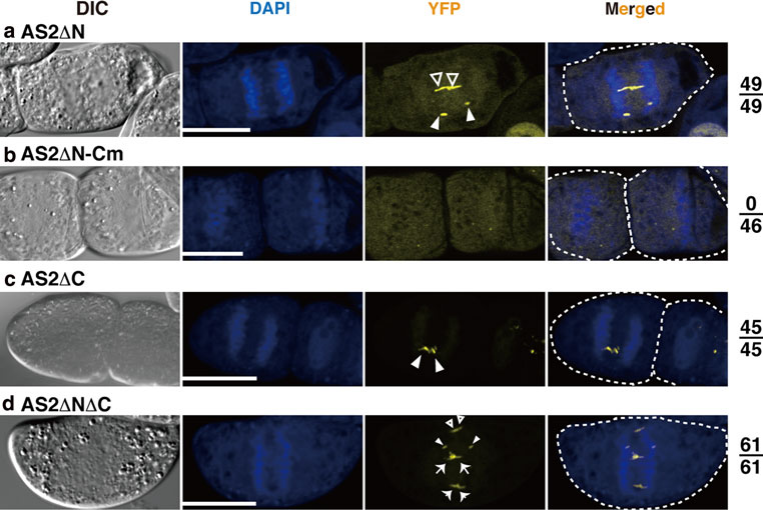
Subcellular localization of deletion mutants of AS2 in M phase cells of transformed BY-2 lines. BY-2 cells harboring each mutant construct were incubated for 16 h in the presence of 0.05 μM 17-β-estradiol. Cells were fixed and stained with DAPI. Fluorescence of DAPI (*blue*) and fluorescence of YFP (*yellow*) in the M-phase cells are visualized by using confocal fluorescence microscopy. Nomarski images (DIC) and merged images of YFP and DAPI (Merged) are shown in the *left panels* and the *right panels*, respectively. *Numbers on the right* represent ratios of cells showing AS2 bodies to total M phase cells examined. In anaphase and telophase cells, AS2 bodies that seem to belong to the same pair are marked with a common shape of *arrowheads* or *arrows*. *Bars* 20 μm

In the preceding sections, we have shown that the N-terminal short sequence and the C-motif have negative and positive roles, respectively, in the formation of AS2 bodies in cells at interphase. We addressed how mutations of these AS2 regions affect the formation of AS2 bodies in cells during mitosis. In cells expressing *AS2ΔN*-*YFP*, *AS2ΔC*-*YFP*, and *AS2ΔNΔC*-*YFP*, pairs of YFP signals were detected in AS2 bodies in all mitotic cells we examined (Fig. 3a, c, d), which suggests that the N-terminal short sequence and the C-terminal half are not required for the formation of AS2 bodies in mitotic cells. Distinct speckles were not observed in cells expressing *AS2ΔN*-*Cm*-*YFP*, however, even during mitosis (Fig. 3b). These results show that the C-motif is essential for the formation of AS2 bodies in cells during mitosis as well as in interphase.

## Discussion

### AS2 bodies are consistently formed in cells during mitosis

In the present study, we have shown that AS2 bodies were detected in all BY-2 cells undergoing mitosis and in a small proportion of interphase cells. In mitotic cells, the AS2 bodies consistently existed in a pairwise fashion during the segregation of chromosomes (Figs. 1, 3). This suggests that the separation of the AS2 bodies might not be a random behavior but appears instead to be coupled with the segregation of sets of daughter chromosomes. In addition, the average number of AS2 bodies in cells increased by approximately two-fold during the segregation (see the first section of “Results”). Therefore, there might be a mechanism by which the number of AS2 bodies could multiply and at least some amounts of AS2 proteins could be inevitably distributed into daughter cells after cell division. Our results, however, did not clearly show that the AS2 bodies were physically associated with segregating chromosomes. Further examinations are required for understanding how the bodies could be precisely distributed into daughter nuclei.

In addition to M phase cells, a small proportion of interphase cells also contained AS2 bodies, which is consistent with a previous report (Zhu et al. 2008). Most interphase cells (approximately 90%) did not form AS2 bodies. Since the AS2 bodies were associated with the nucleolus, which is present in cells in interphase but disappears in cells during mitosis (Leung et al. 2004), the formation of AS2 bodies might be related to such turnover of nucleoli. It is worth investigating whether the presence of AS2 bodies in interphase cells might be related to a specific phase or status in interphase of the cell cycle.

We have previously reported that when AS1-GFP and AS2-YFP proteins are synthesized in leaf cells of *A. thaliana*, both proteins are co-localized to the AS2 bodies (Ueno et al. 2007). When AS1-YFP and AS2-CFP are synthesized in BY-2 cells, signals from AS1-YFP are detected in both nucleoplasm and nucleolar speckles in interphase cells and in some metaphase cells (Zhu et al. 2008), which is consistent with our present and previous data (Ueno et al. 2007).

In the present study, we used tobacco BY-2 cells to investigate the formation of AS2 bodies. It has not yet been examined whether AS2 bodies in BY-2 cells might have the same or similar structural and functional characteristics as the bodies formed in the Arabidopsis epidermis (Ueno et al. 2007). Our recent results of the deletion analysis of AS2, however, showed that the C-motif was required for the formation of AS2 bodies both in the mitotic cells of BY-2 and in Arabidopsis epidermal cells (our unpublished data), suggesting that both bodies might have similar molecular characteristics. The consistent formation and biological relevance of the AS2 bodies at mitosis should be investigated by further experimentations with Arabidopsis plants.

### The N-terminal half of AS2 protein is crucial for its functions in leaf morphogenesis and the formation of AS2 bodies

The N-terminal short sequence has an inhibitory function in the formation of AS2 bodies, whereas the C-terminal half has no significant effect on AS2 body formation (Fig. 2b). Deletion of the N-terminal region (AS2ΔN) yielded a markedly elevated ability to form the AS2 bodies, but no ability to restore abnormalities of the *as2*-*1* mutant (our unpublished result). These results imply that the N-terminal sequence plays an essential role in the formation of proper leaf shape and that the highly efficient formation of the AS2 bodies has a negative effect on proper leaf morphogenesis. On the other hand, the deletion including both the N-terminal region and the C-motif (AS2ΔN-Cm) abolished the abilities to form AS2 bodies and to rescue the mutant phenotypes of *as2*-*1*. Our results of experiments with both deletion mutants suggest that proper regulation of the formation of AS2 bodies by these regions is crucial for functions of the AS2 protein in leaf morphogenesis.

### AS2 bodies exhibit unique characteristics as nuclear speckles

Cajar bodies are nuclear speckles that dynamically move throughout the cell cycle of animal cells (Sleeman et al. 2003). As shown in the present study, the AS2 bodies consistently existed in BY-2 cells undergoing mitosis and were distributed in a pairwise manner after metaphase, which seems to be different from the dynamic random behavior of Cajal bodies, suggesting that the AS2 bodies are distinct from the Cajar bodies. This suggestion, however, should be confirmed by experiments with marker proteins for the Cajar bodies in cells of *A. thaliana*.

In human cells, the foci of lamin B1, the major B-type lamin protein, are associated with chromosomes during mitosis, but their separation does not take place in a pairwise fashion (Martin et al. 2010). To our knowledge, the mode of distribution for the AS2 bodies should be the first example for speckles that are distributed in a pairwise manner to daughter nuclei during the mitotic progression, although a number of nuclear speckles have been characterized (Bernardi and Pandolfi 2007; Matera et al. 2009; Krieghoff-Henning and Hofmann 2008; Pollock and Huang 2010). Such dynamic behavior of the AS2 protein might be critical for transmission of this protein through cell divisions that are associated with leaf morphogenesis.

In the present studies, we used the YFP-fused AS2 cDNA, which was genetically functional because it restored the *as2* mutant to a wild-type phenotype. Although examinations of endogenous AS2 proteins would be ideal to approach an in vivo status of this protein, markedly low amounts of AS2 in cells of *A. thaliana* make it difficult to clearly visualize sub-cellular distribution of the AS2 protein.

In conclusion, AS2 protein forms speckles designated as AS2 bodies during mitosis of tobacco BY-2 cells. These bodies distribute in a pairwise fashion after metaphase, which suggests that they play a role in transmitting AS2 proteins to daughter cells during cell division and that this process is controlled at least partially by signals encoded by the AS2 sequence itself.

## Electronic supplementary material

Below is the link to the electronic supplementary material.

Supplementary material 1 (DOC 29 kb)

Fig. S1 Primer pairs used for cloning full-length and deletion mutants of AS2. The schematic representation of each gene and the primer pairs used for PCR-amplification are illustrated. The sequences of each primer are shown in Table S1 (TIFF 1579 kb)
