# Supplementary material for: Arabidopsis ASYMMETRIC LEAVES2 protein required for leaf morphogenesis consistently forms speckles during mitosis of tobacco BY-2 cells via signals in its specific sequence
Source: J Plant Res. 2012 Feb 17;125(5):661–8. doi: 10.1007/s10265-012-0479-5 (PMC3428529; doi:10.1007/s10265-012-0479-5)
Supplement: Supplementary file 1 — Supplementary material 1 (DOC 29 kb) [file 10265_2012_479_MOESM1_ESM.doc]

Table S1. Primers used in this study.

| Primer name | Sequence |
| --- | --- |
| #125 | 5’-GGGGCTCGAGATGGCATCTTCTTCAACAAACTC-3' |
| #126 | 5'-TTCCCCATGGCGCCACCTCCGAGCTCAGATTTAGCACAGC-3' |
| #127 | 5'-GGGGCTCGAGATGGTATTCGCGCCCTATTTC-3' |
| #128 | 5'-TTCCCCATGGCGCCACCTCCAGACGGATCAACAGTACGG-3' |
| #129 | 5’-GGGGCTCGAGATGTCACCATGCGCCGCTTG-3' |
