# Supplementary figures and images for: Arabidopsis ASYMMETRIC LEAVES2 protein required for leaf morphogenesis consistently forms speckles during mitosis of tobacco BY-2 cells via signals in its specific sequence
Source: J Plant Res. 2012 Feb 17;125(5):661–8. doi: 10.1007/s10265-012-0479-5 (PMC3428529; doi:10.1007/s10265-012-0479-5)

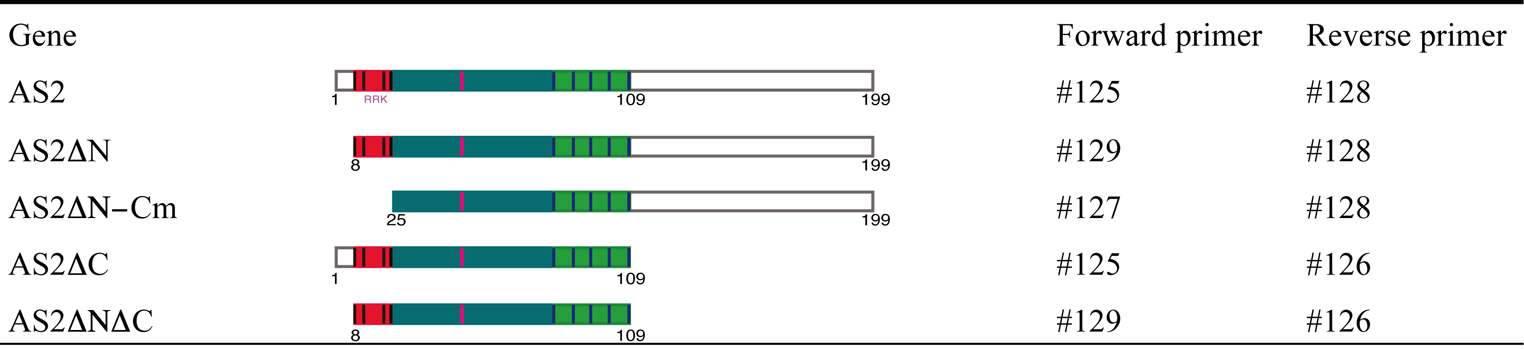

Supplement: Supplementary file 2 — Fig. S1 Primer pairs used for cloning full-length and deletion mutants of AS2. The schematic representation of each gene and the primer pairs used for PCR-amplification are illustrated. The sequences of each primer are shown in Table S1 (TIFF 1579 kb) [file 10265_2012_479_MOESM2_ESM.tif]
